# Efficacy and Safety of Dipeptidyl Peptidase-4 Inhibitors in Type 2 Diabetes Mellitus Patients with Moderate to Severe Renal Impairment: A Systematic Review and Meta-Analysis

**DOI:** 10.1371/journal.pone.0111543

**Published:** 2014-10-31

**Authors:** Dongsheng Cheng, Yang Fei, Yumei Liu, Junhui Li, Yuqiang Chen, Xiaoxia Wang, Niansong Wang

**Affiliations:** Department of Nephrology and Rheumatology, Shanghai Jiaotong University Affiliated Sixth People's Hospital, Shanghai, P.R. China; University Medical Center Utrecht, Netherlands

## Abstract

**Objective:**

To perform a systematic review and meta-analysis regarding the efficacy and safety of dipeptidyl peptidase-4 (DDP-4) inhibitors (“gliptins”) for the treatment of type 2 diabetes mellitus (T2DM) patients with moderate to severe renal impairment.

**Methods:**

All available randomized-controlled trials (RCTs) that assessed the efficacy and safety of DDP-4 inhibitors compared with placebo, no treatment, or active drugs were identified using PubMed, EMBASE, Cochrane CENTRAL, conference abstracts, clinical trials.gov, pharmaceutical company websites, the FDA, and the EMA (up to June 2014). Two independent reviewers extracted the data, and a random-effects model was applied to estimate summary effects.

**Results:**

Thirteen reports of ten studies with a total of 1,915 participants were included in the final analysis. Compared with placebo or no treatment, DPP-4 inhibitors reduced HbA1c significantly (−0.52%, 95%CI −0.64 to −0.39) and had no increased risk of hypoglycemia (RR 1.10, 95%CI 0.92 to 1.32) or weight gain. In contrast to glipizide monotherapy, DPP-4 inhibitors showed no difference in HbA1c lowering effect (−0.08%, 95% CI −0.40 to 0.25) but had a lower incidence of hypoglycemia (RR 0.40, 95%CI 0.23 to 0.69). Furthermore, DPP-4 inhibitors were well-tolerated, without any additional mortality and adverse events. However, the quality of evidence was mostly as low, as assessed using the GRADE system for each outcome.

**Conclusions:**

DPP-4 inhibitors are effective at lowering HbA1c in T2DM patients with moderate to severe renal impairment. DPP-4 inhibitors also have a potential advantage in lowering the risk of adverse events. Regarding the low quality of the evidence according to GRADE, additional well-designed randomized trials that focus on the safety and efficacy of DPP-4 inhibitors in various CKD stages are needed urgently.

## Introduction

The prevalence of type 2 diabetes mellitus (T2DM) and chronic kidney disease (CKD) is increasing steadily. Diabetes is the leading cause of CKD, which might progress to end-stage renal disease and increase the risk of death [1]. It is known that good glycemic control might delay the deterioration in kidney function [2]. However, antihyperglycemic therapy, including the use of metformin, sulfonylureas, thiazolidinediones, and insulin, in T2DM patients with renal impairment remains controversial regarding its tolerability and safety. Metformin might no longer be the first choice for CKD patients because of the risk of lactic acidosis. The KDIGO recommended that metformin could be continued in individuals with a glomerular filtration rate (GFR)>45 ml/min/1.73 m^2^, should be reviewed in those with a GFR 30–44 ml/min/1.73 m^2^, and should be discontinued in patients with a GFR<30 ml/min/1.73 m^2^ [3]. Selected sulfonylureas are associated with a higher risk of hypoglycemia, which can be worse in CKD patients [4]. For thiazolidinediones, although there is no higher risk of hypoglycemia or dose adjustment in renal failure patients, they might cause fluid retention and edema, which are common manifestations of kidney disease [5]. Although insulin is used widely, its dose has to be adjusted occasionally based on blood glucose to avoid hypoglycemia because it is partially metabolized by the kidney [6].

Dipeptidyl peptidase-4 (DPP-4) inhibitors are a novel type of oral glucose-lowering agents that modulate fasting plasma glucose, postprandial glucose, and HbAlc levels by decreasing the inactivation of incretins such as glucagon-like peptide 1 and glucose-dependent insulinotropic polypeptide to stimulate the release of insulin in a glucose-dependent manner [7]–[8]. Since most DPP-4 inhibitors are eliminated by the kidney, a dose reduction is required for patients with moderate to severe renal impairment, except for linagliptin because of its relatively reduced renal metabolism [9]–[10]. Giorda et al [9] conducted a systematic review on the pharmacokinetics, safety, and efficacy of DPP-4 inhibitors in patients with both T2DM and renal impairment, and suggested that DDP-4 inhibitors could be appropriate drugs for patients with renal impairment. However, their study lacked sufficient randomized trials; therefore, a further meta-analysis is needed.

The aim of the current study was to perform a systematic review and meta-analysis of the safety and efficacy of DPP-4 inhibitors for the treatment of T2DM patients with moderate to severe renal impairment.

## Methods

This review was conducted and reported according to PRISMA (Preferred Reporting Items for Systematic Reviews and Meta-Analysis; Table S1) [11].

### Search Strategy

Literature searches were performed using PubMed, EMBASE, and Cochrane CENTRAL to identify studies published before June 20, 2014, with no language restrictions. The main search term was a combination of MESH terms and text words for DPP-4 inhibitors and renal impairment. The details of the search are presented in File S1. To find additional relevant studies, a full search was conducted of the references lists of the identified studies and abstracts from the 2011 to 2013 annual meetings of the American Diabetes Association and the European Association for the Study of Diabetes. Additional unpublished trials were searched from clinicaltrials.gov (www.clinicaltrials.gov) and relevant pharmaceutical company websites. Finally, the Food and Drug Administration (FDA; www.fda.gov) and European Medicines Agency (EMA; www.ema.europa.eu) websites were searched for medical reviews of DPP-4 inhibitors (alogliptin, linagliptin, saxagliptin, and sitagliptin in the FDA, and vildaligptin in the EMA).

### Eligibility criteria for the inclusion in the meta-analysis

The inclusion criteria were as follows: (1) randomized controlled trials, (2) duration ≥12 weeks, (3) studies assessing T2DM patients with moderate or severe renal insufficiency, including dialysis patients, and (4) a comparison of DPP-4 inhibitors with placebo, no treatment, or other active drugs.

The degree of renal impairment was classified as non-dialysis patients including moderate renal insufficiency (estimated glomerular filtration rate [eGFR] 30–60 ml/min or ml/min/1.73 m^2^), severe renal insufficiency (eGFR<30 ml/min or ml/min/1.73 m^2^, not on dialysis), and persons receiving dialysis.

### Study selection

The titles, abstracts, and/or full-text were assessed independently by DC and YL using the abovementioned inclusion criteria. Discrepancies were resolved by consensus, and the reasons for exclusion were recorded. Endnote X4 was used for literature management and selection.

### Data extraction

Data were extracted independently by DC and YL using electronic extraction forms. The extracted data included the authors, study title, publication year, study design, number of participants, mean age of the participants, follow-up duration, completeness, inclusion criteria, baseline HbA1C, intervention type, intervention dose, sponsor information, and pre-specified outcomes. The main outcome for efficacy was the mean change in HbA1c from baseline. Other efficacy outcomes included the mean changes in glycated albumin, fasting blood glucose (FPG), 2-h postprandial glucose, and the proportion of participants that achieved the goal of an HbA1c<7%. The parameters used to assess safety included the incidence of hypoglycemia, the mean change in body weight and renal function, the incidence of cardiovascular disease events, mortality, the incidence of any adverse events (AE), serious adverse events (SAE), drug-related AE, drug-related SAE, and the discontinuation rate. In the current meta-analysis, data from the longest follow-up report were extracted. For studies that contained a 12-week placebo treatment as the initial control and an extended 40–42-week sulfonylurea treatment, only the data from the first period were extracted.

### Risk of bias assessment and grading the quality of the evidence

The risk of bias was evaluated using the Cochrane Collaboration's “risk of bias” tool [12]. The overall risk of bias was classified into three grades: low risk of bias if all bias domains were low, high risk if any bias domain was high, and the remaining cases were judged as unclear. The quality of evidence for each outcome was assessed using Grading of Recommendations Assessment, Development, and Evaluation (GRADE) [13], and GRADEprofiler was used to create a GRADE evidence profile (Version 3.6.1, GRADE Working Group).

### Data analysis and synthesis

The mean difference (MD) and its 95% confidence interval (CI) were applied for continuous variables, whereas the risk ratio (RR) and its 95%CI were used for dichotomous outcomes. All analyses were based on a random-effects model. Statistical heterogeneity across trials was analyzed using χ^2^ tests (*p*<0.10) and *I* ^2^ tests [14]. *I* ^2^ values of 25%, 50%, and 75% corresponded to low, medium, and high levels of heterogeneity, respectively [15]. Subgroup analyses were performed to investigate potential sources of heterogeneity (such as participants, interventions, and study quality). Sensitivity analyses were performed to assess changes in HbA1c by excluding reports at high risk of bias, eliminating unpublished reports, removing open label studies, and using fixed effects models. Publication bias for the main outcome was assessed primarily using funnel plots. Egger's and Begg's tests were applied to provide statistical evidence of funnel graph symmetry. In addition, the trim and fill method was applied to evaluate the influence of missing studies. Meta analyses were performed using Review Manager Software (Version 5.1. The Cochrane Collaboration, 2011). Funnel plots, Egger's and Begg's tests, and the trim and fill method were performed using Stata software (Version 11, College Station, TX).

## Results

### Search results and study characteristics

**Figure 1:**
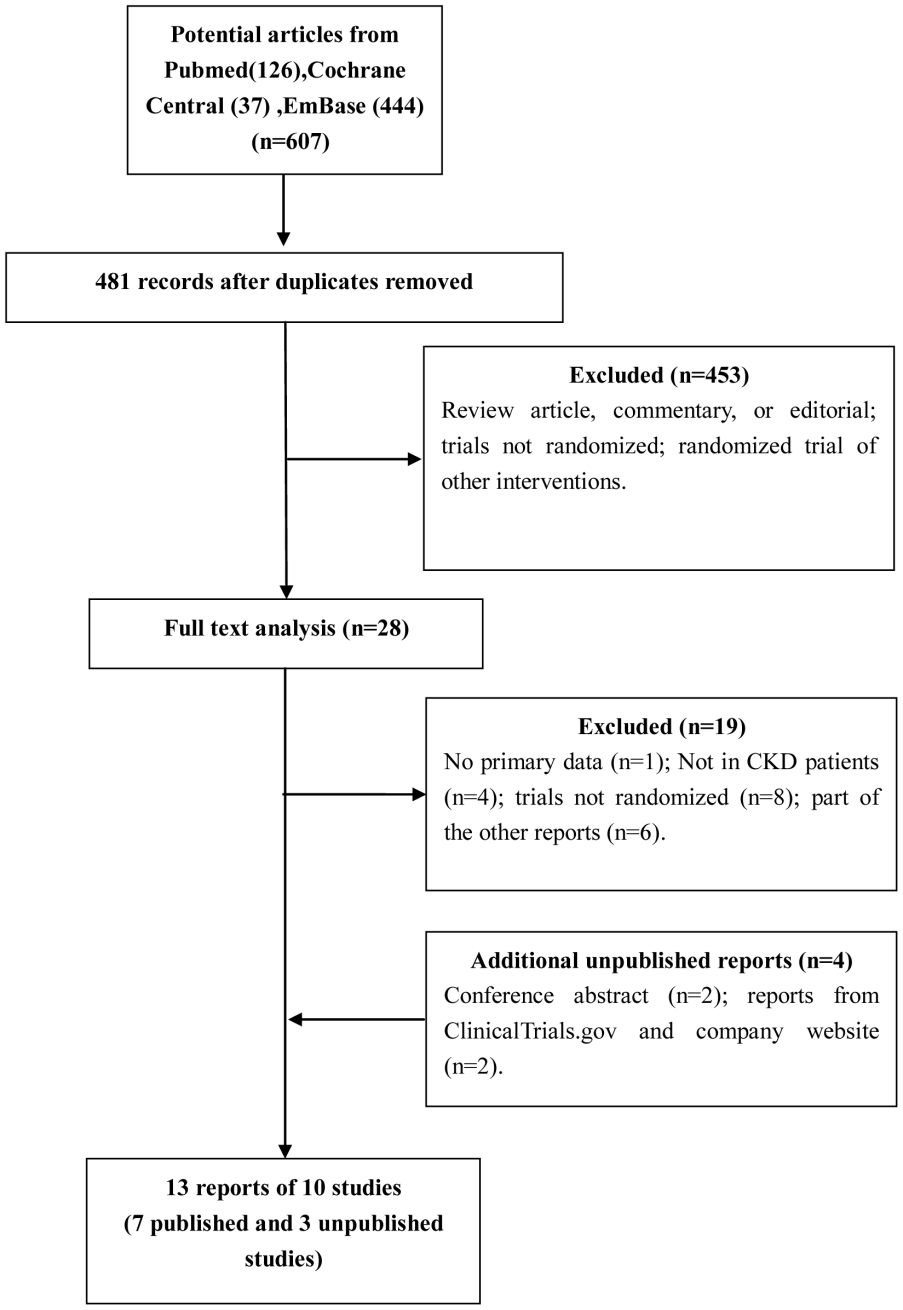
Study flow diagram for trial selection and exclusion.

Figure 1 shows the flow diagram of trial selection. From the initial 607 records in the electronic databases, a total of 28 articles were examined in full, among which nine reports on seven studies (seven primary reports and two extensions) [16]–[24] were finally selected. Furthermore, four additional unpublished reports (three primary reports and one extension) [25]–[28] obtained from conference abstracts, ClinicalTrials.gov and company website were selected, yet one trial on gemigliptin conducted in October 2013 identified in ClinicalTrials.gov was excluded [29]. Ultimately, thirteen reports on ten RCT studies were included in this systematic review.

The characteristics of the included trials are presented in Table 1. With the exception of one open label study, all trials were designed as double-blinded parallel studies and included a total of 1,915 participants; the enrollment sizes ranged from 51–369 individuals. The reports were published between 2008 and 2013, with durations of 12–54 weeks. The mean baseline HbA1c varied from 6.7% to 8.5%.

**Table 1:** Characteristics of the randomized controlled clinical trials included in this analysis.

Trial	Design	Extension periods	Participant details			Type of prevention		Follow-up details	
			Total number	Baseline HbA1C	Renal status	I	C	Duration	Completeness
**DPP-4 inhibitor vs. placebo or no treatment**									
Chan, 2008 [16] *	Double blinded RCT	NA	91	I, 7.6% vs. C, 7.8%	eGFR<50 ml/min, including HD and PD patients	Sitagliptin (25–50 mg/day)+SBT	Placebo+SBT	12 weeks	I, 70.8% vs. C, 76.9%
Ito, 2011 [17]	Open label RCT	NA	51	I, 6.7% vs. C, 6.7%	HD patients	Vildagliptin (50–100 mg/day)+SBT	SBT	24 weeks	I, 100% vs. C, 100%
Nowicki, 2011a [18]	Double blinded RCT	Nowicki 2011b [19]	170	I, 8.5% vs. C, 8.1%	eGFR<50 ml/min, including HD patients	Saxagliptin (2.5 mg/day)+SBT	Placebo+SBT	52 weeks (12+40)	I, 49% vs. C, 58%
Lukashevich, 2011 [20]	Double blinded RCT	Kothny 2012 [21]	369	I, 7.8% vs. C, 7.8%	eGFR<50 ml/min/1.73 m^2^, including HD patients	Vildagliptin (50 mg/day)+SBT	Placebo+SBT	52 weeks (24+28)	I, 67.8% vs. C, 60.8%
McGill, 2013 [22]	Double blinded RCT	NA	133	I, 8.2% vs. C, 8.2%	eGFR<30 ml/min/1.73 m^2^, not requiring dialysis	Linagliptin (5 mg/day)+SBT	Placebo+SBT	52 weeks	I, 72.1% vs. C, 73.8%
Laakso, 2013 [23] *	Double blinded RCT	NA	235	I, 8.1% vs. C, 8.0%	eGFR<60 ml/min/1.73 m^2^, not requiring dialysis	Linagliptin (5 mg/day)+SBT	Placebo+SBT	12 weeks	NR
**DPP-4 inhibitor vs. glipizide**									
Ferreira, 2013a [24]	Double blinded RCT	NA	422	I, 7.8% vs. C, 7.8%	eGFR<50 ml/min/1.73 m^2^, not requiring dialysis	Sitagliptin (25–50 mg/day)+glipizide matching placebo	Glipizide (2.5–20 mg/day)+sitagliptin matching placebo	54 weeks	I, 77.7% vs. C, 80.2%
Ferreria, 2013b [25]	Double blinded RCT	NA	129	I, 7.9% vs. C, 7.8%	HD and PD patients	Sitagliptin (25 mg/day)+glipizide matching placebo	Glipizide (2.5–20 mg/day)+sitagliptin matching placebo	54 weeks	I, 73% vs. C, 69%
**DPP-4 inhibitor vs. GLP-1 receptor agonist albiglutide**									
Leiter, 2013 [26] #	Double blinded RCT	NA	231	I, 8.2% vs. C, 8.2%	eGFR 15–60 ml/min/1.73 m^2^	Sitagliptin (25–100 mg/day)+albiglutide matching placebo+SBT	Albiglutide (30 mg/week)+sitagliptin matching placebo+SBT	26 weeks	NR
**Vildagliptin vs. sitagliptin**									
Novartis, 2011a [27]	Double blinded RCT	Novartis, 2011b [28]	84	NR	eGFR<30 ml/min/1.73 m^2^	Vildagliptin (50 mg/day)+sitagliptin matching placebo+SBT	Sitagliptin (25 mg/day)+vildagliptin matching placebo+SBT	52 weeks (24+28)	NR

I, DPP-4 inhibitor group; C, control group; NA, not applicable; NR, not reported; HD, hemodialysis; PD, peritoneal dialysis; eGFR, estimated glomerular filtration rate; SBT, stable background therapy.

*For studies that used placebo as the comparison group for the first period of time (12 weeks) and sulfonylureas for an extended 40–42 weeks, only data from the first period were extracted for the meta-analyses.

# Only data for subjects with moderate to severe renal impairment in this trial were extracted for analysis.

Among the enrolled articles, six studies (or eight reports) focused mainly on non-dialysis patients, two assessed dialysis patients, and two (or three reports) analyzed both types of patients. The study by Kothny et al. [21], which contained only two hemodialysis subjects, was included in the non-dialysis group. The studies also differed in the type of DPP-4 inhibitors used: five (or six reports) on sitagliptin, three (or four reports) on vildagliptin, two on linagliptin, and one (or two reports) on saxagliptin.

Four studies (or six reports) compared DPP-4 inhibitors with placebo or no treatment in patients who were already treated with a stable antidiabetic therapy [17]–[22]. Two studies compared DPP-4 inhibitor monotherapy with glipizide monotherapy [24]–[25]. In addition, two studies used placebo as a control for the first period (12 weeks) followed by an extended 40–42-week sulfonylurea treatment, which was ignored because of the potential influence of the first period [16], [23]. The final two studies also compared two different types of DPP-4 inhibitors [27]–[28] and compared the DPP-4 inhibitor with GLP-1 receptor agonist [26].

### Risk of bias in the included studies and quality of the evidence

**Figure 2:**
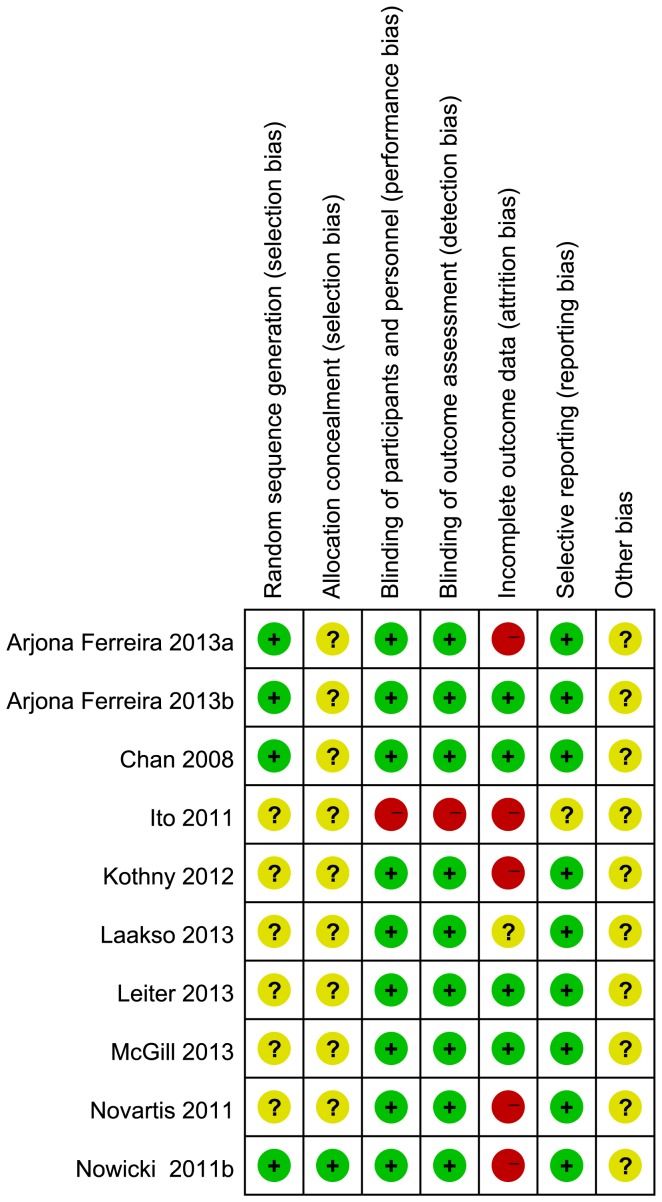
Risk of bias summary of the included studies.

Random sequence generation was adequate in four of the ten trials (40%). The allocation concealment was stated clearly in one trial (10%). Nine trials (90%) blinded the participants, investigators, and outcome assessors. The outcome data were incomplete in five trials (50%), and selective reporting was not found. The loss to follow-up rate ranged from 0 to 51%. The risk of bias domains of the included studies are shown in Figures 2 and S1. The quality of evidence assessed using the GRADE approach ranged from moderate for any AE to low or very low for all other outcomes (Files S2 and S3).

### Glycemic efficacy

#### HbA1c

**Figure 3:**
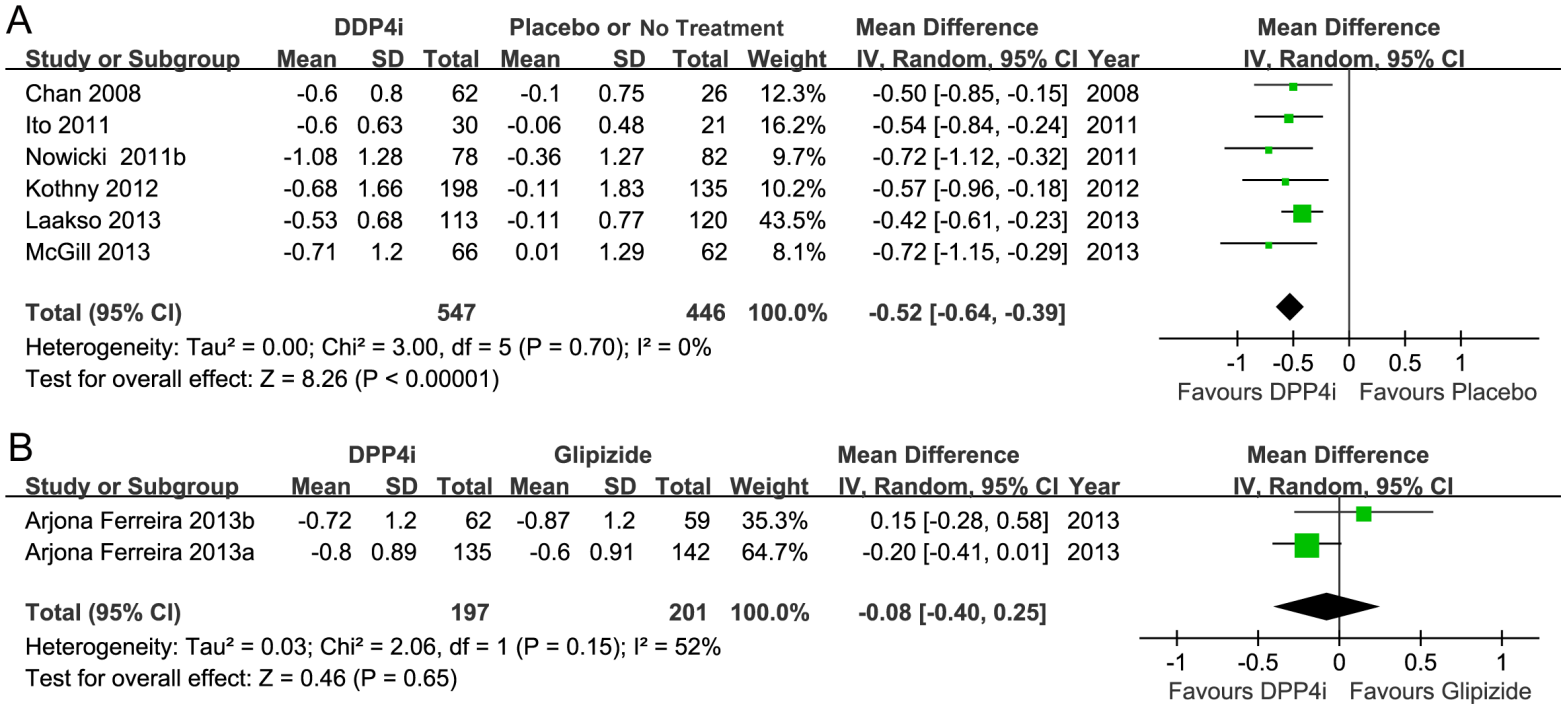
Meta-analysis for changes in HbA1c levels. A, DPP-4 inhibitor vs. placebo or no treatment. B, DPP-4 inhibitor vs. glipizide.

**Figure 4:**
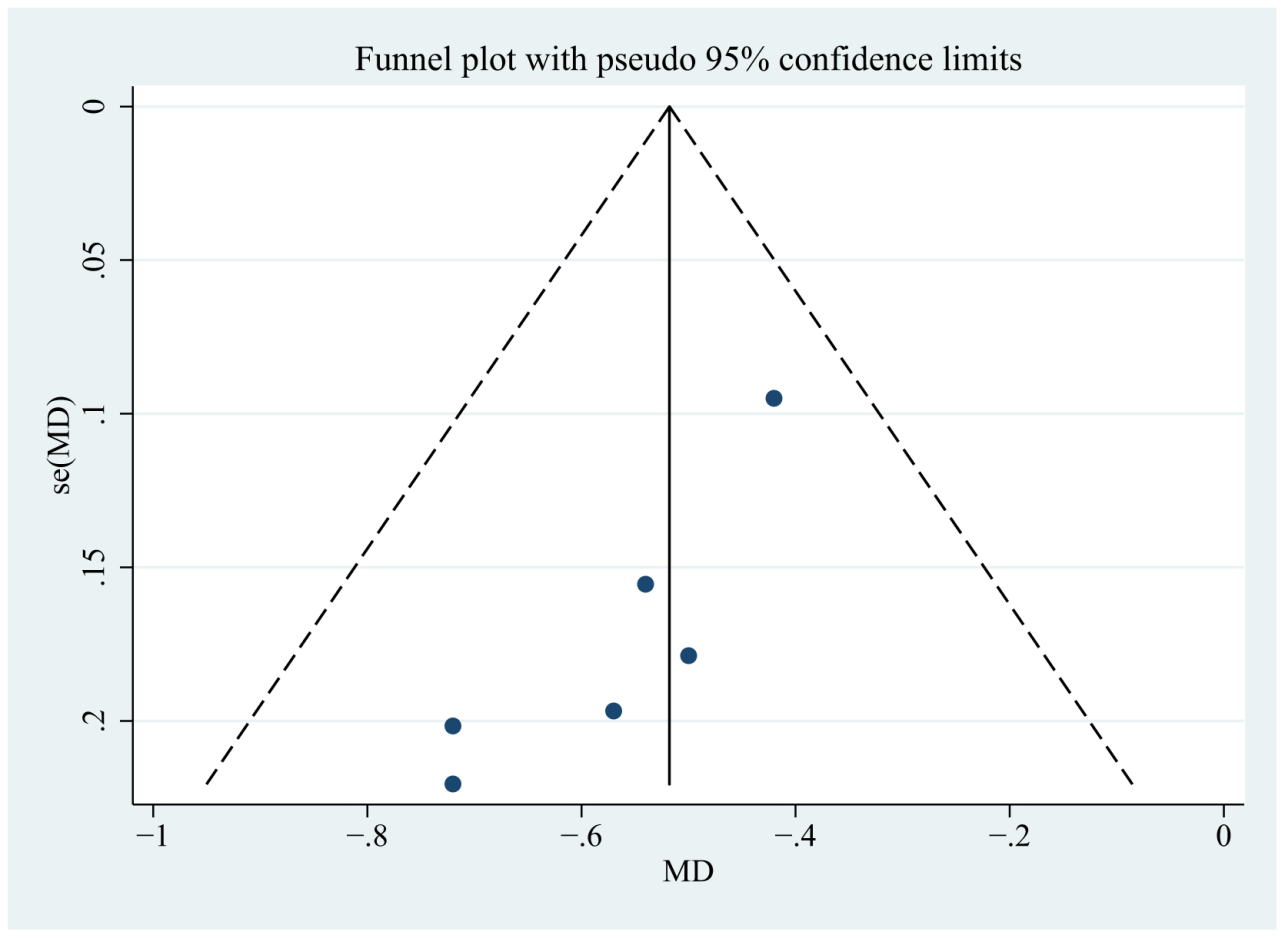
Funnel plot for mean difference (MD) in the change in HbA1c (DPP-4 inhibitor vs. placebo or no treatment).

The efficacy of the DPP-4 inhibitors was analyzed thoroughly and compared with the placebo or no treatment group. A statistically significant improvement was observed in HbA1c in individuals treated with DPP-4 inhibitors (random MD −0.52, 95%CI −0.64 to −0.39; *p*<0.00001) without heterogeneity (*I* ^2^ = 0%; *p* = 0.70) (Figure 3A). The HbA1c change was not significantly different in groups with varied dialysis statuses (test for subgroup differences *p* = 0.94). The mean change was −0.50 in the non-dialysis group, compared with −0.49 in the dialysis group (Figure S2). Sensitivity analyses were performed to assess the change in HbA1c by excluding reports at a high risk of bias, eliminating unpublished reports, removing open-label studies, and using fixed-effects models. The results revealed that all the effect sizes were similar in magnitude and direction to the overall estimates (Table S2). The funnel plot (Figure 4) for this outcome was asymmetrical, which was confirmed using Egger's and Begg's tests (*p* = 0.013 and *p* = 0.039, respectively). The trim and fill method was also used to evaluate the influence of the possible missing studies. However, no trimming was performed and the data were unchanged.

According to the two trials (398 participants) that compared monotherapy using DPP-4 inhibitor or glipizide, there was no significant difference in HbA1c reduction between groups (random MD −0.08, 95%CI −0.40 to 0.25; *p* = 0.65). The heterogeneity was moderate (*I* ^2^ = 52%; *p* = 0.15) (Figure 3B). A subgroup analysis in another study that compared the efficacy of sitagliptin and albiglutide revealed a significant difference in HbA1c reduction in moderate renal insufficiency patients (−0.53, 95%CI −0.80 to −0.26), whereas no difference was found in the severe renal insufficiency group (−0.47, 95%CI −1.12 to 0.18).

#### Responder rates: achieving an HbA1c of <7%

**Figure 5:**
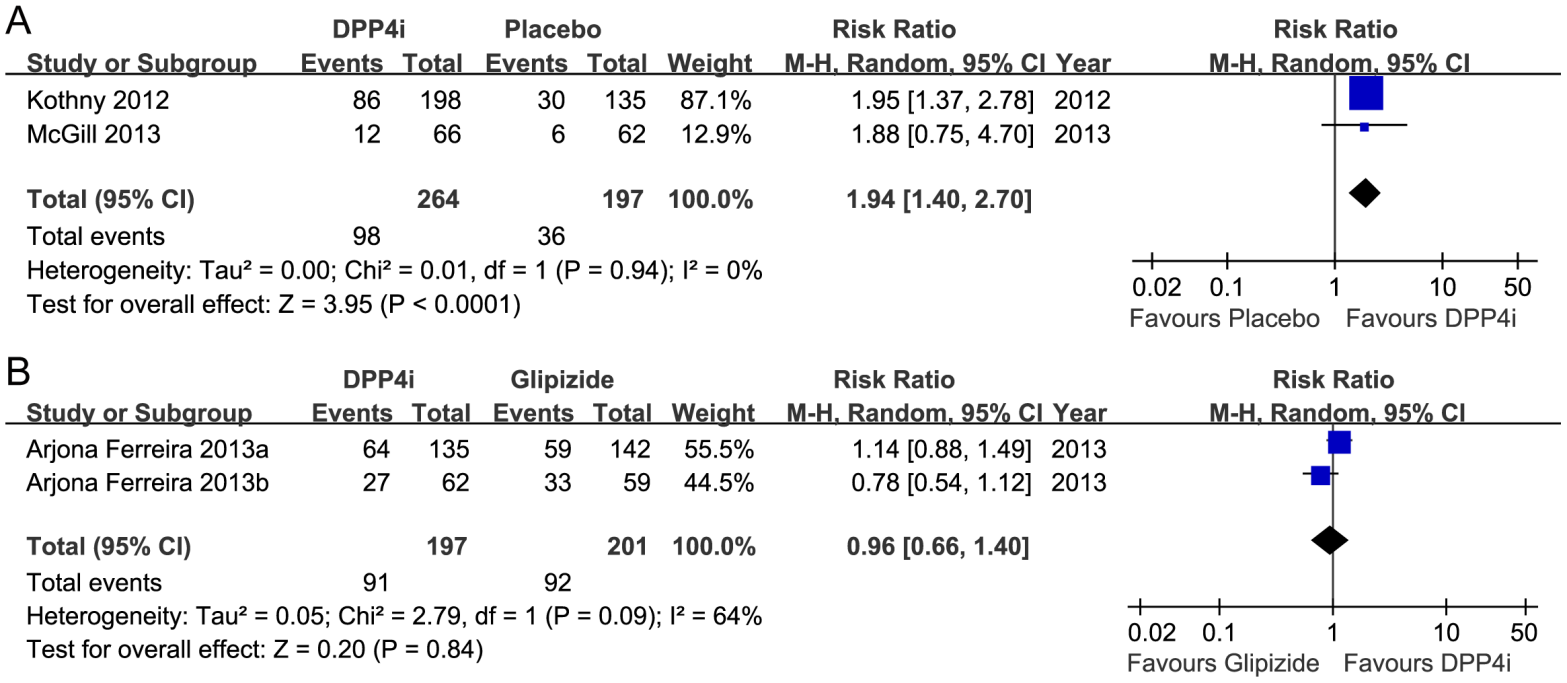
Risk ratio for achieving an HbA1c<7%. A, DPP-4 inhibitor vs. placebo. B, DPP-4 inhibitor vs. glipizide.

Four studies (838 participants) used achieving an HbA1c target of <7% as an outcome. Compared with the placebo or no treatment groups, the DPP-4 inhibitor groups had a higher proportion of patients who achieved the HbA1c goal (461 participants, random RR 1.94, 95%CI 1.40 to 2.70; *p*<0.0001). Heterogeneity was absent (*I* ^2^ = 0%; *p* = 0.94) (Figure 5A).

There was no difference in the proportion of patients receiving glipizide monotherapy and DPP-4 inhibitor monotherapy that achieved an HbA1c <7% (random RR 0.96, 95%CI 0.66 to 1.40; *p* = 0.84); the heterogeneity was moderate (*I* ^2^ = 64%; *p* = 0.09) (Figure 5B).

#### Fasting blood glucose (FBG)

**Figure 6:**
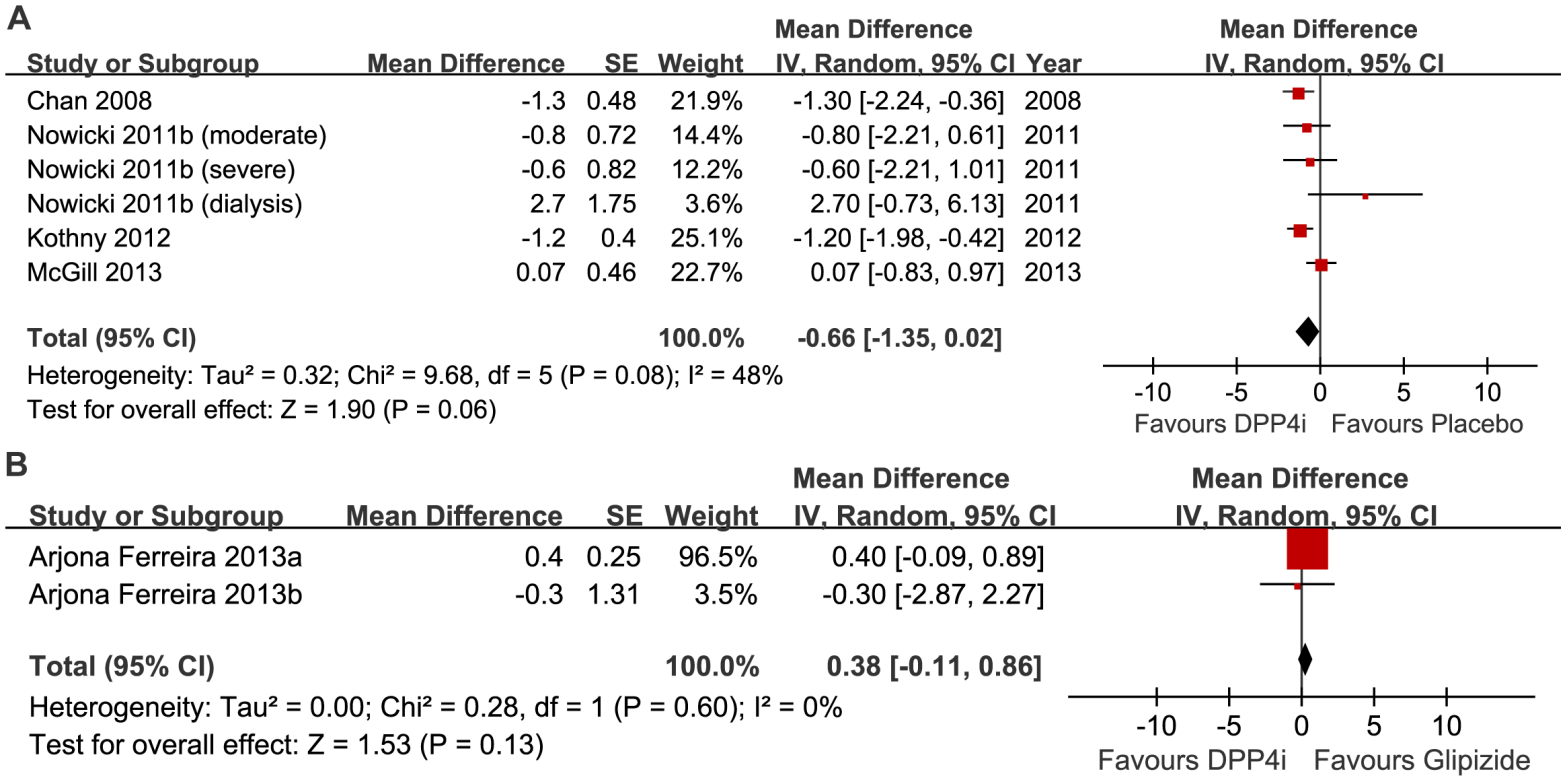
Meta-analysis for the change in fasting blood glucose. A, DPP-4 inhibitor vs. placebo. B, DPP-4 inhibitor vs. glipizide.

Changes in FBG from baseline were described in six studies. As shown in Figure 6A, there was no significant decrease in FBG between the DPP-4 inhibitor and placebo groups, and the heterogeneity was moderate (522 participants, random MD −0.66, 95%CI −1.35 to 0.02, *p* = 0.06; *I* ^2^ = 48%). The result remained consistent after excluding reports at a high risk of bias (random MD −0.61, 95%CI −1.95 to 0.73, *p* = 0.37; *I* ^2^ = 76%), and dialysis patients (random MD −0.64, 95%CI −1.30 to 0.01, *p* = 0.05; *I* ^2^ = 32%). There was also no significant difference between DPP-4 inhibitor monotherapy and glipizide monotherapy (random MD 0.38, 95%CI −0.11 to 0.86; *p* = 0.13); there was no heterogeneity (*I* ^2^ = 0%; *p* = 0.60) (Figure 6B).

#### Glycated albumin and 2-h postprandial glucose

Only one study (51 dialysis participants) that compared a DPP-4 inhibitor with a no treatment group reported glycated albumin and 2-h postprandial glucose. After 24 weeks of intervention with vildagliptin, the mean glycated albumin levels were decreased significantly from 24.5% at baseline to 20.5%, and the mean postprandial glucose levels decreased from 186 mg/dl at baseline to 140 mg/dl (all *p*<0.0001) [17].

### Safety

#### Body weight

**Figure 7:**
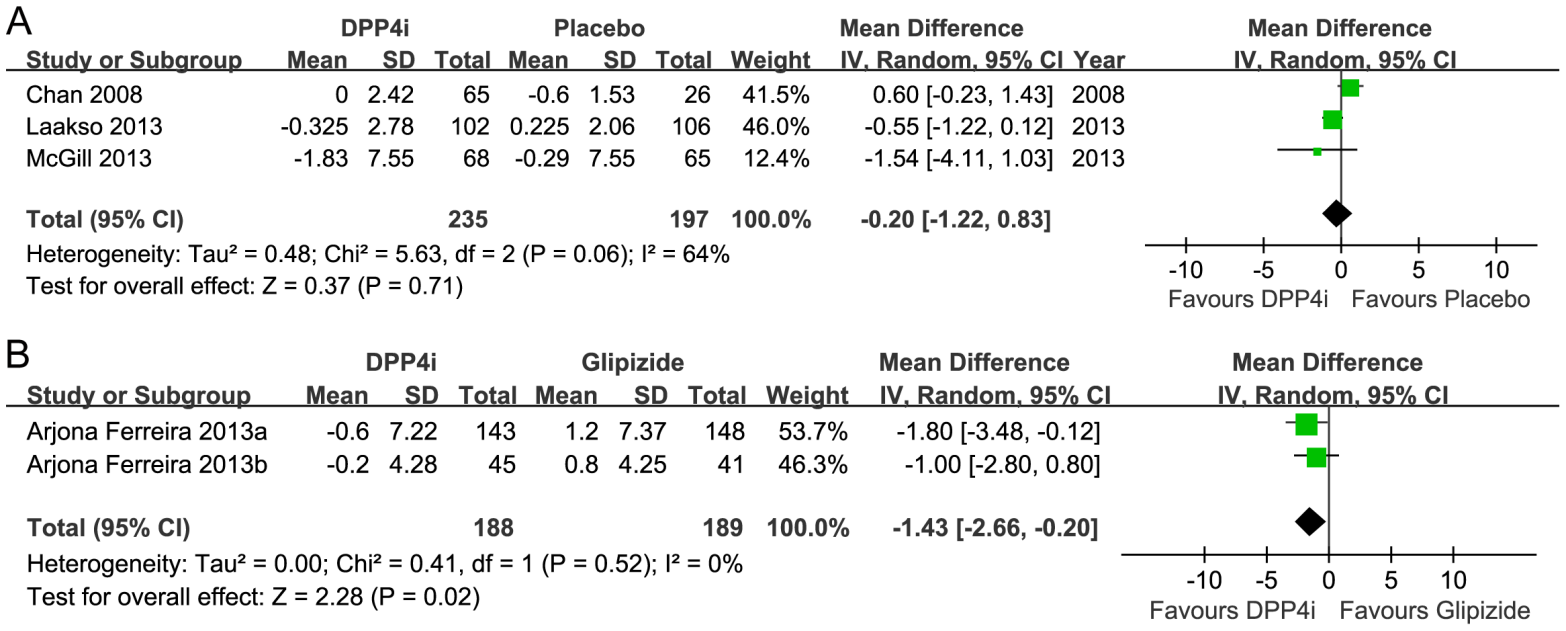
Meta-analysis for changes in bodyweight. A, DPP-4 inhibitor vs. placebo. B, DPP-4 inhibitor vs. glipizide.

Seven trials reported changes in body weight, but only five trials (809 participants) were included in the pooled analysis. Compared with the placebo group, the DPP-4 inhibitor group had a neutral weight profile (432 participants, random MD −0.20, 95%CI −1.22 to 0.83; *p* = 0.71). The heterogeneity was moderate (*I* ^2^ = 64%; *p* = 0.06) (Figure 7A). There were no significant differences in the non-dialysis group (341 participants; random MD −0.61, 95%CI −1.26 to 0.03; *p* = 0.06). There was no heterogeneity (*I* ^2^ = 0%; *p* = 0.46). The dialysis group was excluded from the analysis because of its effect on dry weight.

DPP-4 inhibitor, but not glipizide monotherapy, treatment resulted in decreased body weight (377 participants, random MD −1.43, 95%CI −2.66 to −0.20; *p* = 0.02). There was no heterogeneity (*I* ^2^ = 0%; *p* = 0.52) (Figure 7B).

#### Hypoglycemia

**Figure 8:**
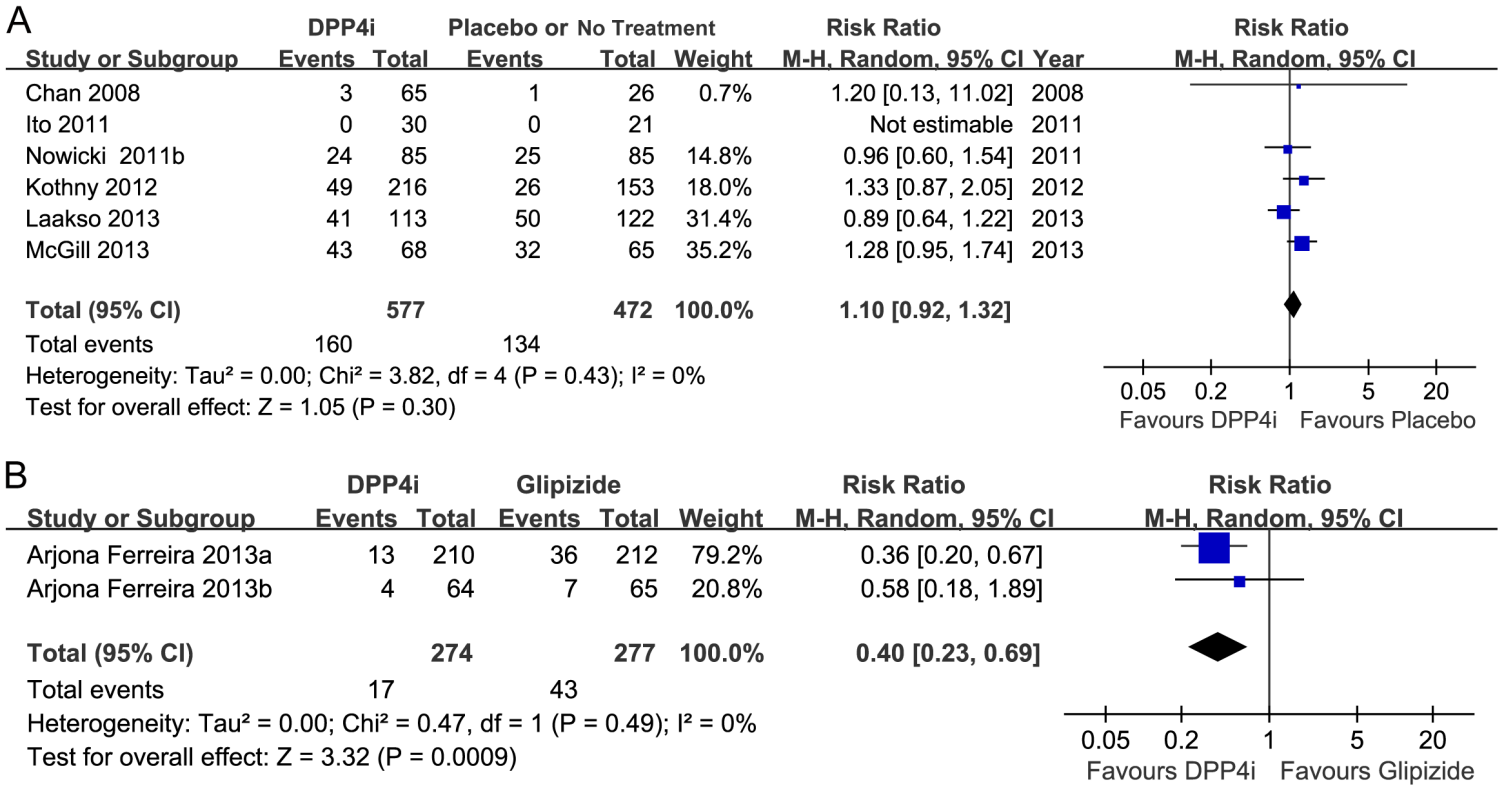
Meta-analysis for the risk of hypoglycemia. A, DPP-4 inhibitor vs. placebo. B, DPP-4 inhibitor vs. glipizide.

All studies reported the incidence of hypoglycemia, and seven of 10 studies also mentioned severe hypoglycemia; eight cases occurred in the DPP-4 inhibitor groups (464 participants), and ten occurred in the placebo groups (350 participants). Compared with placebo or no treatment, DPP-4 inhibitor therapy did not increase the incidence of hypoglycemia (1,049 participants, random RR 1.10, 95%CI 0.92 to 1.32; *p* = 0.30) (Figure 8A), and there was no heterogeneity (*I* ^2^ = 0%; *p* = 0.43). Subgroup analyses according to dialysis status were not performed due to insufficient data.

Only two articles compared the effects of DPP-4 inhibitor and glipizide on hypoglycemia. Data revealed that the DPP-4 inhibitor group had a lower incidence of hypoglycemia than did the glipizide group (551 participants, random RR 0.40, 95%CI 0.23 to 0.69; *p* = 0.0009; there was no heterogeneity (*I* ^2^ = 0%; *p* = 0.42) (Figure 8B). Compared with glipizide, the DPP-4 inhibitor reduced the incidence of severe hypoglycemia in dialysis patients significantly (five of 64 participants vs. 0 of 64, respectively) but not in non-dialysis patients (six cases of 212 participants vs. three cases of 210 participants). In contrast, there was no significant difference between vildagliptin and sitagliptin regarding their risk of causing hypoglycemia (0 cases of 46 participants vs. two of 38 participants, respectively).

#### Renal function and cardiovascular disease events

Seven studies assessed the relationship between DPP-4 inhibitors and renal function in non-dialysis patients using several parameters such as changes in eGFR (four studies), serum creatinine (one study), and the progression of renal status (two studies). Although four of the studies focused on a change in eGFR, a reliable pooled estimate could not be performed due to the varied objects used for comparison and a lack of accurate standard deviation values. Nevertheless, the individual studies showed no obvious effect of DPP-4 inhibitors on renal function.

Five studies (1,137 participants) reported cardiovascular disease (CVD) events. However, because different definitions of CVD events were used, a pooled estimate was not available. Table S3 shows the number of participants who experienced CVD events in each trial. There was no difference in CVD incidence between the DPP-4 inhibitor and control groups.

#### Total mortality and adverse events

Most studies reported total mortality and the incidence of adverse events, as summarized in Table 2. There was no obvious difference in mortality, the incidence of adverse events (any AE, SAE, drug-related AE, and drug-related SAE), and discontinuation rate.

**Table 2:** Summary of safety outcomes for DPP-4 Inhibitors compared to comparators.

Outcome	Number of studies	Total number	Events	RR (95%CI)
**Death**				
PP-4 inhibitor vs. placebo	5	512/446	10/8	1.12 (0.44–2.85)
PP-4 inhibitor vs. glipizide	2	274/277	7/13	0.54 (0.22–1.33)
ildagliptin vs. sitagliptin	1	46/38	0/0	NA
**Discontinuation**				
PP-4 inhibitor vs. placebo	4	512/446	38/33	1.02 (0.65–1.60)
PP-4 inhibitor vs. glipizide	2	274/277	23/25	0.93 (0.54–1.60)
ildagliptin vs. sitagliptin	1	46/38	6/4	1.24 (0.38–4.07)
**Any AE**				
PP-4 inhibitor vs. placebo	4	482/425	397/342	1.01 (0.95–1.08)
PP-4 inhibitor vs. glipizide	2	274/277	196/205	0.97 (0.87–1.07)
**SAE**				
PP-4 inhibitor vs. placebo	4	482/425	105/94	0.97 (0.76–1.23)
PP-4 inhibitor vs. glipizide	2	274/277	57/58	0.99 (0.72–1.37)
ildagliptin vs. sitagliptin	1	46/38	15/10	1.24 (0.63–2.43)
**Drug-related AE**				
PP-4 inhibitor vs. placebo	3	397/350	113/105	0.96 (0.77–1.20)
PP-4 inhibitor vs. glipizide	2	274/277	37/52	0.72 (0.49–1.06)
**Drug-related SAE**				
PP-4 inhibitor vs. placebo	2	198/207	8/7	1.16 (0.43–3.10)
PP-4 inhibitor vs. glipizide	2	274/277	2/1	2.02(0.18–22.10)

AE, adverse event; SAE, serious adverse event; NA, not applicable.

## Discussion

The current study used a complete search, integration, and analysis of data to perform a systematic assessment regarding the efficacy and safety of DPP-4 inhibitors in T2DM patients with moderate to severe renal impairment. In contrast to the placebo and no treatment groups, the DPP-4 inhibitor groups typically had lower HbA1c levels, and a higher proportion of subjects that achieved the HbA1c goal of <7%; dialysis status did not affect these results. Sensitivity analysis indicated that the results were robust. There was no significant difference between the capacity of DPP-4 inhibitors and other active drugs such as sulfonylureas to reduce HbA1c levels. In contrast, DPP-4 inhibitors did not exhibit an FBG lowering effect, probably due to heterogeneity among the studies and an insufficient number of the samples. These results are consistent with those obtained in T2DM patients in similar studies [30]–[31]. It was reported previously that GLP-1 reduced HbA1c levels to a greater extent than did DPP-4 inhibitors [32]–[33], yet data in this article showed no difference between DPP-4 inhibitors and GLP-1 in severe renal dysfunction while a relatively poorer reducing effect of DPP-4 inhibitors was indeed observed in moderate renal dysfunction. The results might become more consistent with the previous reports if there was a larger sample size.

Regarding safety, data revealed that there was no change in the incidence of hypoglycemia with DPP-4 inhibitor treatment. The results remained consistent with dialysis patients with severe hypoglycemia. Moreover, there was no increase in weight gain, incidence of severe adverse effects, and total mortality. Although the definition of CVD was different among the trials included in this systematic review, no studies reported an increase in the incidence of CVD events as a result of DPP-4 inhibitor therapy. This is consistent with recent evidence showing that DPP-4 inhibitors did not increase CVD events in T2DM patients [34]–[35].

Although individual studies indicated that there was no deterioration in renal status during DPP-4 inhibitor administration, this conclusion could be strengthened by a pooled estimation on renal function. However, this could not be accomplished in the current study due to variations in the comparison objects and a lack of accurate standard deviation values. In addition, the effect of DDP-4 inhibitors on renal function in CKD patients remains unclear. Previous reports found either the progression of renal status or no affect on renal deterioration in patients treated with these agents [36]–[37]. Recently, studies assessing the attenuation of renal dysfunction in an ischemia-reperfusion injury model and remnant kidneys revealed that there might be a potential nephroprotective effect of DPP-4 inhibitors [38]–[39]. The mechanism for this might be associated with a reduction in apoptosis and inflammation and an increase in antioxidant production. Although a large number of ongoing trials have assessed the proteinuria-lowering effect of DPP-4 inhibitors [40]–[41], few primary articles have investigated the effect of these agents on renal status, particularly in individuals at an early stage of renal impairment. Therefore, there is little evidence that DPP-4 inhibitors could help delay the progression of renal status.

This is the first meta-analysis to assess the safety and efficacy of DPP-4 inhibitors in T2DM patients with moderate to severe renal impairment. The strength of this article is that it reports a comprehensive search that enrolled almost all the published and unpublished articles, including conferences abstracts, clinical trials registries, company websites, and FDA and EMA websites. In addition, a transparent GRADE approach provided an accurate assessment of the quality of evidence for each outcome.

However, the study also has some limitations. Notably, no studies at a low risk of bias were found, which could be attributed to several factors. For example, some studies might have inadequate random sequence generation and allocation sequence concealment. Moreover, the included open label study might have affected the results due to the lack of blinding. Articles with incomplete outcome data might have overestimated the efficacy of DPP-4 inhibitors on lowering HbA1c. In addition, all the above studies were sponsored by pharmaceutical companies, which might increase the risk of bias. A summary of the findings and the quality of evidence for this review are shown in Files S2 and S3. Although GRADE analysis indicated that the quality of evidence for each outcome was mostly low, ‘any adverse events’ were graded moderate. Due to insufficient data, our subgroup analyses were based on dialysis status rather than CKD stages. In addition, the dialysis group focused mainly on hemodialysis patients, with few data on peritoneal dialysis. However,it is quite necessary and difficult to establish good glycemic control for peritoneal dialysis patients due to the clinical use of glucose-containing dialysis solution [42]. Therefore, it is important to conduct further testing on the efficacy and safety of DPP-4 inhibitors in patients at various CKD stages and peritoneal dialysis. In addition, more attention should be paid to glycated albumin, which is a new and potentially better indicator of glycemic control, particularly in ESRD patients [43]–[44].

There are several different types of DPP-4 inhibitors, and each lacks sufficient testing. Therefore, we were unable to perform a detailed meta-analysis. The same problem exists for assessing the difference between DPP-4 inhibitors and other active agents, and for subgroup analyses based on different antidiabetic backgrounds.

Funnel plot asymmetry was observed and confirmed using Egger's and Begg's tests. This is recognized as a sign of potential publication bias. However, several alternative factors might also lead to asymmetry [45], including selection bias, poor methodological design, inadequate analysis in smaller studies, and true heterogeneity induced by factors such as different durations of intervention [46]. As shown in Figure 4, three studies in the lower left quadrant contributed to the asymmetry because of a long follow-up period and a high risk of bias (due to the inclusion of incomplete outcome data in two studies). Therefore, the asymmetry in our analysis might be attributed to true heterogeneity caused by clinical and methodological heterogeneity, even though no statistical heterogeneity was detected. Finally, it is inevitable that some unpublished articles might have been missed from the search.

This comprehensive analysis revealed that DPP-4 inhibitors lowered HbA1c, and did not increase the incidence of adverse events. Therefore, they represent a feasible treatment option for T2DM patients with moderate to severe renal impairment. However, considering the overall low quality of the data in this study, as judged by GRADE, DPP-4 inhibitors should be tested further in better-designed randomized trials. Additional attention should be paid to a comparison between DPP-4 inhibitors and other active drugs in patients at different stages of CKD, particularly including dialysis patients.

## Conclusions

DPP-4 inhibitors are effective at lowering HbA1c in T2DM patients with moderate to severe renal impairment, and might lower the risk of adverse events. Further well-designed randomized trials focusing on the safety and efficacy of DPP-4 inhibitors in patients at different CKD stages are needed urgently because of the low quality of evidence in the current meta-analysis.

## Supporting Information

Figure S1 **Risk of bias graph of all included studies.**
(TIF)

Figure S2 **Subgroup analysis for change in HbA1c (DPP-4 inhibitor vs placebo or no treatment).**
(TIF)

Table S1 **PRISMA Checklist.**
(DOC)

Table S2 **Sensitivity analyses comparing DPP-4 inhibitors with placebo on HbA1c.**
(DOC)

Table S3 **Findings of comparing DPP-4 inhibitors with comparators on CVD.**
(DOC)

File S1 **Search strategies.**
(DOC)

File S2 **Summary of findings: Efficacy of DPP-4 inhibitors in type 2 diabetes mellitus patients with moderate to severe renal impairment.**
(DOC)

File S3 **Summary of findings: Safety of DPP-4 inhibitors in type 2 diabetes mellitus patients with moderate to severe renal impairment.**
(DOC)
