# Supplementary figures and images for: Efficacy and Safety of Dipeptidyl Peptidase-4 Inhibitors in Type 2 Diabetes Mellitus Patients with Moderate to Severe Renal Impairment: A Systematic Review and Meta-Analysis
Source: PLoS One. 2014 Oct 31;9(10):e111543. doi: 10.1371/journal.pone.0111543 (PMC4216116; doi:10.1371/journal.pone.0111543)

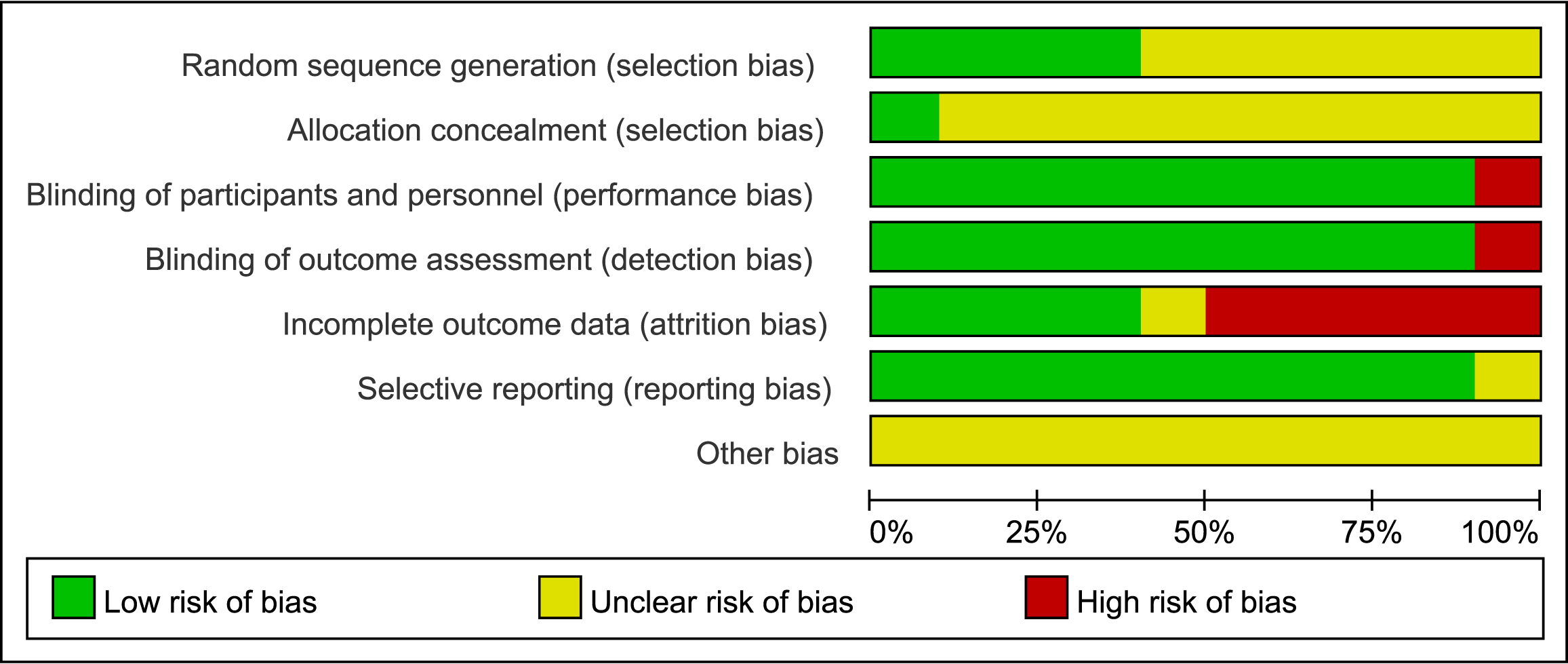

Supplement: Figure S1 — Risk of bias graph of all included studies. (TIF) [file pone.0111543.s001.tif]

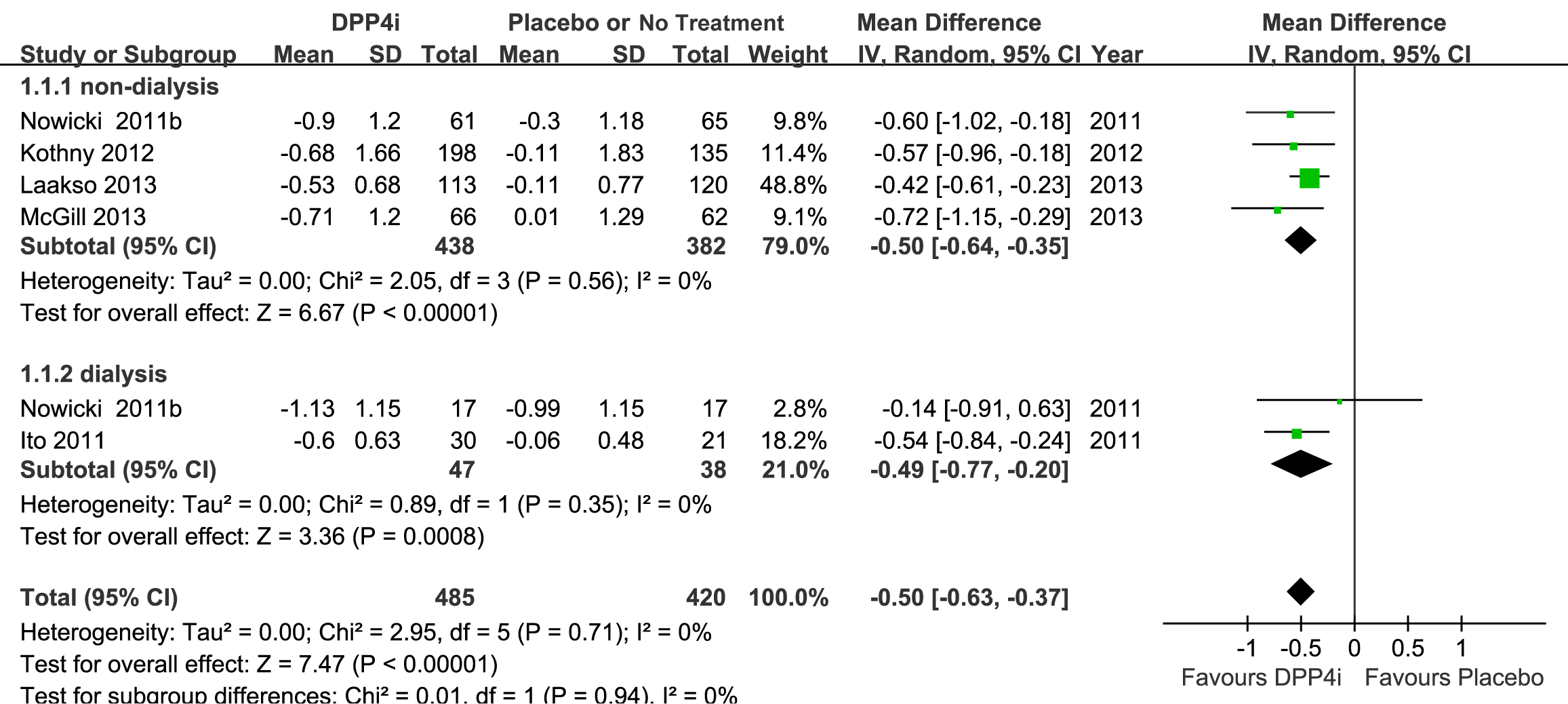

Supplement: Figure S2 — Subgroup analysis for change in HbA1c (DPP-4 inhibitor vs placebo or no treatment). (TIF) [file pone.0111543.s002.tif]
