# Supplementary material for: Efficacy and Safety of Dipeptidyl Peptidase-4 Inhibitors in Type 2 Diabetes Mellitus Patients with Moderate to Severe Renal Impairment: A Systematic Review and Meta-Analysis
Source: PLoS One. 2014 Oct 31;9(10):e111543. doi: 10.1371/journal.pone.0111543 (PMC4216116; doi:10.1371/journal.pone.0111543)
Supplement: Table S2 — Sensitivity analyses comparing DPP-4 inhibitors with placebo on HbA1c. (DOC) [file pone.0111543.s004.doc]

Table S2. Sensitivity analyses comparing DPP-4 inhibitors with placebo on HbA1c.

| **Type of comparison and analysis** | **References** | **DPP-4 inhibitors** | **Placebo or**  **no treatment** | **Mean difference (95% CI)** | **I^2^ (p-value)** |
| --- | --- | --- | --- | --- | --- |
| Excluding unpublished reports | 16-17,19,21-22 | 434 | 326 | -0.59 (-0.76,-0.43) | 0%(P=0.89) |
| In fixed effect inverse variance model | 16-17,19,21-23 | 547 | 446 | -0.52 (-0.62,-0.39) | 0%(P=0.70) |
| Using the report excluding the high risk | 16, 22-23 | 241 | 208 | -0.47(-0.63,-0.32) | 0%(P=0.45) |
| Excluding open label study | 16,19,21-23 | 517 | 425 | -0.51(-0.65,-0.38) | 0%(P=0.56) |
