# Supplementary material for: Efficacy and Safety of Dipeptidyl Peptidase-4 Inhibitors in Type 2 Diabetes Mellitus Patients with Moderate to Severe Renal Impairment: A Systematic Review and Meta-Analysis
Source: PLoS One. 2014 Oct 31;9(10):e111543. doi: 10.1371/journal.pone.0111543 (PMC4216116; doi:10.1371/journal.pone.0111543)
Supplement: Table S3 — Findings of comparing DPP-4 inhibitors with comparators on CVD. (DOC) [file pone.0111543.s005.doc]

Table S3. Findings of comparing DPP-4 inhibitors with comparators on CVD outcome

| **Trial** | **Total number** | **Events** | **RR(95% CI)** | **Definition of CVD** |
| --- | --- | --- | --- | --- |
| **DPP4i vs placebo** |  |  |  |  |
| Kothny 2012 | 216/153 | 30/18 | 1.18(0.68,2.04) | cardiovascular events |
| McGill 2013 | 68/65 | 7/9 | 0.74(0.29,1.88) | cardiovascular death, stroke,  myocardial infarction, and hospitalization  for unstable angina |
| **DPP4i vs glipizide** |  |  |  |  |
| Arjona Ferreira 2013a | 210/212 | 8/11 | 0.73(0.30,1.79) | cardiovascular,cerebrovascular, and peripheralvascular events |
| Arjona Ferreira 2013b | 64/65 | 5/6 | 0.85(0.27,2.63) | cardiovascular,  cerebrovascular, and peripheral  vascular events , heart failure |
| **vildagliptin vs sitagliptin** |  |  |  |  |
| Novartis 2011 | 46/38 | 6/6 | 0.83(0.29,2.35) | Cardiovascular and cerebrovascular event |

**CVD:** cardiovascular disease.
