# Supplementary material for: Efficacy and Safety of Dipeptidyl Peptidase-4 Inhibitors in Type 2 Diabetes Mellitus Patients with Moderate to Severe Renal Impairment: A Systematic Review and Meta-Analysis
Source: PLoS One. 2014 Oct 31;9(10):e111543. doi: 10.1371/journal.pone.0111543 (PMC4216116; doi:10.1371/journal.pone.0111543)
Supplement: File S1 — Search strategies. (DOC) [file pone.0111543.s006.doc]

**PubMed 2014-6-20 126**

#1 Dipeptidyl-Peptidase IV Inhibitors [mh]

#2 Dipeptidyl-Peptidase IV Inhibitor* [tw]

#3 Dipeptidyl-Peptidase 4 Inhibitor* [tw]

#4 dpp-4 [tw]

#5 dpp-iv [tw]

#6 dpp4 [tw]

#7 dppiv [tw]

#8 gliptin* [tw]

#9 sitagliptin [nm]

#10 sitagliptin* [tw]

#11 Linagliptin [nm]

#12 Linagliptin* [tw]

#13 dutogliptin [nm]

#14 dutogliptin* [tw]

#15 alogliptin [nm]

#16 alogliptin* [tw]

#17 vildagliptin [nm]

#18 vildagliptin* [tw]

#19 saxagliptin [nm]

#20 saxagliptin* [tw]

#21 Gemigliptin [nm]

#22 Gemigliptin* [tw]

#23 teneligliptin [nm]

#24 teneligliptin*[tw]

#25 #1 or #2 or #3 or #4 or #5 or #6 or #7 or #8 or #9 or #10 or #11 or #12 or #13 or #14

or #15 or #16 or #17 or #18 or #19 or #20 or #21 or #22 or #23 or #24

# 26 kidney diseases [MH]

# 27 renal insufficiency [TW]

# 28 renal impairment [TW]

# 29 renal failure [TW]

# 30 chronic kidney diseases [TW]

# 31 renal replacement therapy [MH]

# 32 renal dialysis [TW]

# 33 hemodialysis [tw]

# 34 dialysis [tw]

# 35 end stage renal diseases [tw]

# 36 ESRD [TW]

#37 #26 or #27 or #28 or #29 or #30 or #31 or #32 or #33 or #34

or #35 or #36

#38 randomized controlled trial [pt]

#39 controlled clinical trial [pt]

#40 randomized [tiab]

#41 randomised [tiab]

#42 placebo [tiab]

#43 drug therapy [sh]

#44 randomly [tiab]

#45 trial [tiab]

#46 groups [tiab]

#47 #38 or #39 or #40 or #41 or #42 or #43 or #44

or #45 or #46

#48 animals [mh] NOT humans [mh]

#49 #47 NOT #48

#50 #25 AND #37 AND #49

**EMBASE 2014-6-20 444**

#1 exp Dipeptidyl-Peptidase IV Inhibitor/

#2 Dipeptidyl-Peptidase IV Inhibit$.tw

#3 Dipeptidyl-Peptidase 4 Inhibit$.tw

#4 dpp-4.tw

#5 dpp-iv.tw

#6 dpp4.tw

#7 dppiv.tw

#8 $gliptin$.tw

#9 exp sitagliptin/

#10 sitagliptin.tw

#11 exp Linagliptin/

#12 Linagliptin.tw

#13 exp dutogliptin/

#14 dutogliptin.tw

#15exp alogliptin/

#16 alogliptin.tw

#17 exp vildagliptin/

#18 vildagliptin.tw

#19 exp saxagliptin/

#20 saxagliptin.tw

#21 exp Gemigliptin/

#22 Gemigliptin.tw

#23 exp teneligliptin/

#24 teneligliptin.tw

#25 OR #1-24

#26 exp kidney disease

#27 exp renal replacement therapy/

#28 renal insufficiency.tw

#29 renal impairment.tw

#30 renal failure.tw

#31 chronic kidney diseases.tw

#32 hemodialysis .tw

#33 dialysis.tw

#34 end stage renal diseases.tw

#35 ESRD.tw

#36 OR #26-35

#37 Clinical trial/

#38 Randomization/

#39 Randomized controlled trial/

#40 Single blind procedure/

#41 Double blind procedure/

#42 Crossover procedure/

#43 Placebo/

#44 Randomi?ed controlled trial$.tw

#45 Rct.tw.

#46 Random allocation.tw.

#47 Randomly allocated.tw.

#48 Allocated randomly.tw.

#49 (allocated adj2 random).tw.

#50 Single blind$.tw.

#51 Double blind$.tw.

#52 ((treble or triple) adj (blind$)).tw.

#53 Placebo$.tw.

#54 Prospective study/

#55 OR #37-54

#56 Case study/

#57 Case report.tw.

#58 Abstract report/ or letter/

#59 OR #56-58

#60 #55 NOT #59

#61 #25 AND #36 AND #60

**Cochrane Library 2014-6-20 37**

#1MeSH descriptor Dipeptidyl-Peptidase IV Inhibitors explode all trees

#2 dpp next iv

#3 dpp next 4

#4 dipeptidyl peptidase iv

#5 dipeptidyl peptidase 4

#6 DPPIV

#7 dpp4

#8 *gliptin*

#9 sitagliptin*

#10 Linagliptin*

#11 dutogliptin*

#12 alogliptin*

#13 vildagliptin*

#14 saxagliptin*

#15 Gemigliptin*

#16 teneligliptin*

#17 #1 or #2 or #3 or #4 or #5 or #6 or #7 or #8 or #9 or #10 or #11 or #12 or #13 or #14

or #15 or #16

#18 MeSH descriptor Kidney Diseases explode all trees

#19 MeSH descriptor Renal Replacement Therapy explode all trees

#20 renal insufficiency

#21 renal impairment

#22 renal failure

#23 chronic kidney diseases

#24 hemodialysis

#25 dialysis.tw

#26 end stage renal diseases

#27 ESRD

#28 #18 or #19 or #20 or #21 or #22 or #23 or #24 or #25 or #26 or #27

#29 #27AND #28
