# Supplementary material for: Efficacy and Safety of Dipeptidyl Peptidase-4 Inhibitors in Type 2 Diabetes Mellitus Patients with Moderate to Severe Renal Impairment: A Systematic Review and Meta-Analysis
Source: PLoS One. 2014 Oct 31;9(10):e111543. doi: 10.1371/journal.pone.0111543 (PMC4216116; doi:10.1371/journal.pone.0111543)
Supplement: File S2 — Summary of findings: Efficacy of DPP-4 inhibitors in type 2 diabetes mellitus patients with moderate to severe renal impairment. (DOC) [file pone.0111543.s007.doc]

| **Efficacy of DPP-4 inhibitors in type 2 diabetes mellitus patients with moderate to severe renal impairment** | | | | | | |
| --- | --- | --- | --- | --- | --- | --- |
| **Patient or population:** type 2 diabetes mellitus patients with moderate to severe renal impairment **Settings:**  **Intervention:** DPP4i | | | | | | |
| **Outcomes** | **Illustrative comparative risks* (95% CI)** | | **Relative effect (95% CI)** | **No of Participants (studies)** | **Quality of the evidence (GRADE)** | **Comments** |
|  | Assumed risk | Corresponding risk |  |  |  |  |
|  | **Control** | **DPP4i** |  |  |  |  |
| **Change in HbA1c from baseline** (DDP4i vs Placebo or no treatment) | The mean change in hba1c from baseline ranged across control groups from  **-0.36 to 0.1** | The mean change in hba1c from baseline in the intervention groups was **0.52 lower** (0.64 to 0.39 lower) |  | 993 (6 studies) | ⊕⊕⊝⊝ **low**^1,2^ |  |
| **Change in HbA1c from baseline**  (DPP4i vs Glipizide) | The mean change in hba1c from baseline ranged across control groups from  **-0.87 to -0.6** | The mean change in hba1c from baseline in the intervention groups was **0.08 lower** (0.4 lower to 0.25 higher) |  | 398 (2 studies) | ⊕⊝⊝⊝ **very low**^1,2,3^ |  |
| **A1C Responder rate**  (DPP4i vs Placebo) | **183 per 1000** | **355 per 1000** (256 to 493) | **RR 1.94**  (1.4 to 2.7) | 461 (2 studies) | ⊕⊝⊝⊝ **very low**^1,2,4^ |  |
| **A1C Responder rate**  (DPP4i vs Glipizide) | **458 per 1000** | **439 per 1000** (302 to 641) | **RR 0.96**  (0.66 to 1.4) | 398 (2 studies) | ⊕⊝⊝⊝ **very low**^1,2,3,4^ |  |
| **Change in FBG from baseline**  (DDP4i vs Placebo) | The mean change in fbg from baseline ranged across control groups from  **-1.06 to 2.73** | The mean change in fbg from baseline in the intervention groups was **0.66 lower** (1.35 lower to 0.02 higher) |  | 528 (4 studies) | ⊕⊝⊝⊝ **very low**^2,3,5^ |  |
| **Change in FBG from baseline**  (DPP4i vs Glipizide) | The mean change in fbg from baseline ranged across control groups from  **-1.73 to -1.33** | The mean change in fbg from baseline in the intervention groups was **0.38 higher** (0.11 lower to 0.86 higher) |  | 397 (2 studies) | ⊕⊕⊝⊝ **low**^1,2^ |  |
| *The basis for the **assumed risk** (e.g. the median control group risk across studies) is provided in footnotes. The **corresponding risk** (and its 95% confidence interval) is based on the assumed risk in the comparison group and the **relative effect** of the intervention (and its 95% CI).  **CI:** Confidence interval; **RR:** Risk ratio; | | | | | | |
| GRADE Working Group grades of evidence **High quality:** Further research is very unlikely to change our confidence in the estimate of effect.  **Moderate quality:** Further research is likely to have an important impact on our confidence in the estimate of effect and may change the estimate. **Low quality:** Further research is very likely to have an important impact on our confidence in the estimate of effect and is likely to change the estimate. **Very low quality:** We are very uncertain about the estimate. | | | | | | |
| ^1^ incomplete outcome data ^2^ too few studies ^3^ I2 value is large  ^4^ small sample size  ^5^ 95% confidence interval includes no effect and crosses the minimal important difference | | | | | | |
