# Supplementary material for: Efficacy and Safety of Dipeptidyl Peptidase-4 Inhibitors in Type 2 Diabetes Mellitus Patients with Moderate to Severe Renal Impairment: A Systematic Review and Meta-Analysis
Source: PLoS One. 2014 Oct 31;9(10):e111543. doi: 10.1371/journal.pone.0111543 (PMC4216116; doi:10.1371/journal.pone.0111543)
Supplement: File S3 — Summary of findings: Safety of DPP-4 inhibitors in type 2 diabetes mellitus patients with moderate to severe renal impairment. (DOC) [file pone.0111543.s008.doc]

| **Safety of DPP-4 inhibitors in patients with type 2 diabetes mellitus and moderate to severe renal impairment** | | | | | | |
| --- | --- | --- | --- | --- | --- | --- |
| **Patient or population:** type 2 diabetes mellitus patients with moderate to severe renal impairment **Settings:**  **Intervention:** DPP4i | | | | | | |
| **Outcomes** | **Illustrative comparative risks* (95% CI)** | | **Relative effect (95% CI)** | **No of Participants (studies)** | **Quality of the evidence (GRADE)** | **Comments** |
|  | Assumed risk | Corresponding risk |  |  |  |  |
|  | **Control** | **DPP4i** |  |  |  |  |
| **Hypoglycemia**  (DPP4i vs Placebo) | **284 per 1000** | **312 per 1000** (261 to 375) | **RR 1.1**  (0.92 to 1.32) | 1049 (6 studies) | ⊕⊕⊝⊝ **low**^1,2^ |  |
| **Hypoglycemia**  (DPP4i vs Glipizide) | **155 per 1000** | **62 per 1000** (36 to 107) | **RR 0.4**  (0.23 to 0.69) | 551 (2 studies) | ⊕⊕⊝⊝ **low**^2,3^ |  |
| **Weight gain**  (DPP4i vs placebo) | The mean weight gain ranged across control groups from  **-0.60 to 0.23** | The mean weight gain in the intervention groups was **0.2 lower** (1.22 lower to 0.83 higher) |  | 432 (3 studies) | ⊕⊕⊝⊝ **low**^2,4^ |  |
| **weight gain** (DPP4i vs Glipizide) | The mean weight gain ranged across control groups from  **0.8 to 1.2** | The mean weight gain in the intervention groups was **1.43 lower** (2.66 to 0.2 lower) |  | 377 (2 studies) | ⊕⊝⊝⊝ **very low**^2,3,5^ |  |
| **Death** ( DPP4i vs placebo) | **18 per 1000** | **20 per 1000** (8 to 51) | **RR 1.12**  (0.44 to 2.85) | 958 (5 studies) | ⊕⊕⊝⊝ **low**^2,3^ |  |
| **Death** ( DPP4i vs Glipizide) | **47 per 1000** | **25 per 1000** (10 to 62) | **RR 0.54**  (0.22 to 1.33) | 552 (2 studies) | ⊕⊕⊝⊝ **low**^2,3^ |  |
| **SAE**  (DPP4i vs Placebo) | **221 per 1000** | **215 per 1000** (168 to 272) | **RR 0.97**  (0.76 to 1.23) | 907 (4 studies) | ⊕⊕⊝⊝ **low**^2,3^ |  |
| **SAE** (DPP4i vs Glipizide) | **209 per 1000** | **207 per 1000** (151 to 287) | **RR 0.99**  (0.72 to 1.37) | 551 (2 studies) | ⊕⊕⊝⊝ **low**^2,3^ |  |
| **Drug related AE**  (DPP4i vs Placebo) | **300 per 1000** | **288 per 1000** (231 to 360) | **RR 0.96**  (0.77 to 1.2) | 747 (3 studies) | ⊕⊕⊝⊝ **low**^2,3^ |  |
| **Drug related AE**  (DPP4i vs Glipizide) | **188 per 1000** | **135 per 1000** (92 to 199) | **RR 0.72**  (0.49 to 1.06) | 551 (2 studies) | ⊕⊕⊝⊝ **low**^2,3^ |  |
| **Drug related SAE**  (DPP4i vs Placebo) | **34 per 1000** | **39 per 1000** (15 to 105) | **RR 1.16**  (0.43 to 3.1) | 405 (2 studies) | ⊕⊕⊝⊝ **low**^2,3^ |  |
| **Drug related SAE**  (DPP4i vs Glipizide) | **4 per 1000** | **7 per 1000** (1 to 80) | **RR 2.02**  (0.18 to 22.1) | 551 (2 studies) | ⊕⊕⊝⊝ **low**^2,3^ |  |
| **Any AE**  (DPP4i vs Placebo) | **805 per 1000** | **813 per 1000** (764 to 869) | **RR 1.01**  (0.95 to 1.08) | 907 (4 studies) | ⊕⊕⊕⊝ **moderate**^2^ |  |
| **Any AE**  (DPP4i vs Glipizide) | **740 per 1000** | **718 per 1000** (644 to 792) | **RR 0.97**  (0.87 to 1.07) | 551 (2 studies) | ⊕⊕⊕⊝ **moderate**^2^ |  |
| **discontinuation**  (DPP4i vs Placebo) | **78 per 1000** | **79 per 1000** (50 to 124) | **RR 1.02**  (0.65 to 1.6) | 907 (4 studies) | ⊕⊕⊝⊝ **low**^2,3^ |  |
| **discontinuation**  (DPP4i vs Glipizide) | **90 per 1000** | **84 per 1000** (49 to 144) | **RR 0.93**  (0.54 to 1.6) | 551 (2 studies) | ⊕⊕⊝⊝ **low**^2,3^ |  |
| *The basis for the **assumed risk** (e.g. the median control group risk across studies) is provided in footnotes. The **corresponding risk** (and its 95% confidence interval) is based on the assumed risk in the comparison group and the **relative effect** of the intervention (and its 95% CI).  **CI:** Confidence interval; **RR:** Risk ratio; | | | | | | |
| GRADE Working Group grades of evidence **High quality:** Further research is very unlikely to change our confidence in the estimate of effect.  **Moderate quality:** Further research is likely to have an important impact on our confidence in the estimate of effect and may change the estimate. **Low quality:** Further research is very likely to have an important impact on our confidence in the estimate of effect and is likely to change the estimate. **Very low quality:** We are very uncertain about the estimate. | | | | | | |
| ^1^ The definition of hypoglycemia varied across trials ^2^ too few studies ^3^ small sample size ^4^ I2 value is large  ^5^ incomplete outcome data | | | | | | |
